# Three New *Trichoderma* Species in Harzianum Clade Associated with the Contaminated Substrates of Edible Fungi

**DOI:** 10.3390/jof8111154

**Published:** 2022-10-31

**Authors:** Zi-Jian Cao, Wen-Tao Qin, Juan Zhao, Yu Liu, Shou-Xian Wang, Su-Yue Zheng

**Affiliations:** 1 School of Landscape and Ecological Engineering, Hebei University of Engineering, Handan 056038, China; 2 Institute of Plant Protection, Beijing Academy of Agriculture and Forestry Sciences, Beijing 100097, China

**Keywords:** Hypocreaceae, *Trichoderma*, phylogeny, morphology, taxonomy

## Abstract

*Trichoderma* is known worldwide as biocontrol agents of plant diseases, producers of enzymes and antibiotics, and competitive contaminants of edible fungi. In this investigation of contaminated substrates of edible fungi from North China, 39 strains belonging to 10 *Trichoderma* species isolated from four kinds of edible fungi were obtained, and three novel species belonging to the Harzianum clade were isolated from the contaminated substrates of *Auricularia heimuer* and *Pholiota adipose*. They were recognized based on integrated studies of phenotypic features, culture characteristics, and molecular analyses of RNA polymerase II subunit B and translation elongation factor 1-α genes. *Trichoderma auriculariae* was strongly supported as a separate lineage and differed from *T. vermifimicola* due to its larger conidia. *Trichoderma miyunense* was closely related to *T. ganodermatigerum* but differed due to its smaller conidia and higher optimum mycelial growth temperature. As a separate lineage, *T. pholiotae* was distinct from *T. guizhouense* and *T. pseudoasiaticum* due to its higher optimum mycelial growth temperature and larger conidia. This study extends the understanding of *Trichoderma* spp. contaminating substrates of edible fungi and updates knowledge of species diversity in the group.

## 1. Introduction

*Trichoderma* Pers. is ubiquitous in various niches and around the world. The genus contains at least eight infrageneric clades, of which the Harzianum clade is one of the largest [1]. According to our investigated statistics, the Harzianum clade consists of more than 95 accepted species, which are morphologically heterogeneous and phylogenetically complicated. They play important roles in agriculture, industry, and other fields and are employed as biocides or biofertilizers for plant growth [2,3,4], act as producers of enzymes and antibiotics, and are endophytic in plants that can resist both physiological stress and pathogen invasion [5,6].

Green mold contamination caused by *Trichoderma* spp. in the cultivation and various growth stages of edible fungi has been one of the biggest biological constraints in the industry since the 1980s [7], with the economic losses accounting for 10–20% of total production [8]. At present, green mold is one of the most devastating diseases in nearly all production areas of cultivated edible fungi due to its high disease incidence and serious economic loss [9,10]. Mycelia of *Trichoderma* spp. show stronger competitiveness than those of edible fungi, and thus they can inhibit mycelial growth or decrease the fruiting rate of edible fungi. Lots of green conidia of *Trichoderma* will gradually cover the contaminated substrates or fruiting bodies, and the contaminated fruiting bodies will eventually shrivel and rot.

In order to better understand the *Trichoderma* species contaminating substrates of edible fungi and preserve biological control resources, substrates of edible fungi contaminated by green mold in North China were investigated, and three undescribed species belonging to the Harzianum clade were found on contaminated substrates of *Auricularia heimuer* and *Pholiota adipose*. Their phylogenetic positions were determined based on sequence analyses of the combined translation elongation factor 1-alpha (*tef1*-*α*) and the second largest nuclear RNA polymerase subunit (*rpb2*) genes. Similarities and differences in morphological characteristics between the new species and their closely related species were investigated and compared in detail.

## 2. Materials and Methods

### 2.1. Isolates and Specimens

Specimens were separately collected from contaminated substrate of edible fungi in North China from 2020 to 2022 (Table S1), and strains were isolated following the method of a previous study [11]. The ex-type strains were deposited in the culture collection of Institute of Plant Protection, Beijing Academy of Agriculture and Forestry Sciences (JZB culture collection).

### 2.2. Morphology and Growth Characterization

For morphological studies, growth rates were determined on three different media: potato dextrose agar (PDA; 200 g potato, 18 g dextrose, 18 g agar, and 1 L distilled water), cornmeal dextrose agar (CMD; 40 g cornmeal, 20 g glucose, 18 g agar, and 1 L distilled water), and synthetic low nutrient agar (SNA; 1 g KH_2_PO_4_, 1 g KNO_3_, 0.5 g MgSO_4_·7H_2_O, 0.5 g KCl, 0.2 g glucose, 0.2 g sucrose, 18 g agar, and 1 L distilled water) at 25, 30, and 35 °C in darkness. Mycelial discs (5 mm diameter) were incubated in Petri dishes (90 mm diameter) with three replicates for each isolate. Colony diameters were measured after 3 days. The time when mycelia entirely covered the surface of the plate and the morphological characteristics of colonies, such as colony appearance, color, and spore production, were recorded [12]. For microscopic morphology, photographs were taken with an Axio Imager Z2 microscope (Carl Zeiss, Jena, Germany). Microscopic characteristics and micromorphological data were examined on the cultures grown on SNA and PDA for 7–9 days at 25 °C.

### 2.3. DNA Extraction, PCR Amplification, and Sequencing

Genomic DNA was extracted from the cultures grown on PDA for 7 days using a plant genomic DNA Kit (DP305, TIANGEN Biotech, Beijing, China). Fragments of *tef1*-*α* and *rpb2* were amplified with the primer pairs EF1-728F [13] and TEF1LLErev [14] and fRPB2-5f/7cr [15], respectively. Each PCR reaction consisted of 12.5 µL Premix Taq™ (TaKaRa, Dalian, China), 1.0 µL of forward primer (10 µM), 1.0 µL of reverse primer (10 µM), 1.5 µL of DNA, and 9 µL of double-sterilized water. Polymerase chain reaction (PCR) conditions followed Zhu and Zhuang [16]. The products were purified and subjected to sequencing on an ABI 3730 DNA sequencer (Applied Biosystems, Bedford, MA, USA) at SinoGenoMax company. Sequences generated from this study and those retrieved from GenBank are listed in Table 1.

### 2.4. Phylogenetic Analyses

Sequences for all isolates generated in this study were blasted against the NCBIs GenBank nucleotide datasets (https://blast.ncbi.nlm.nih.gov/Blast.cgi) and MIST (http://mmit.china-cctc.org/index.php) [17] to obtain an initial identification. To identify the phylogenetic positions of *Trichoderma* species isolated from contaminated substrates of edible fungi, *rpb2* and *tef1*-*α* sequences of all *Trichoderma* species belonging to the Harzianum clade were combined for the analyses, with *T. atroviride* and *T. paratroviride* selected as outgroup taxa. Their sequences of type or ex-type strains based on previous publications were downloaded from NCBI database and assembled using BioEdit 7.0.5.3 [18]. Alignment was generated and converted to nexus files with Clustal X 1.83 [19].

Maximum parsimony (MP) analysis was performed with PAUP 4.0b10. Starting trees were obtained via random stepwise addition with 1000 replicates and subsequent branch-swapping algorithm using tree bisection–reconnection (TBR) [20]. Analyses were performed with all characters treated as unordered and unweighted, and gaps treated as missing data. MaxTrees was set to 1000, and branches collapsed when maximum branch length was zero. Maximum parsimony bootstrap proportion (MPBP) was calculated to test topological confidence of the resulting trees.

Bayesian inference (BI) trees were calculated using MrBayes v. 3.1.2 [21]. The best-fit nucleotide substitution model GTR+I+G was selected using MrModeltest 2.3 [22]. Four chains were run from random trees for 6,000,000 generations and sampled every 100 generations. The first 25% of trees were discarded as the burn-in phase of the analyses, and Bayesian inference posterior probability (BIPP) was determined from the remaining trees. Trees were visualized in FigTree v1.4.3 [23].

## 3. Results

### 3.1. Phylogenetic Analyses

**Figure 1:**
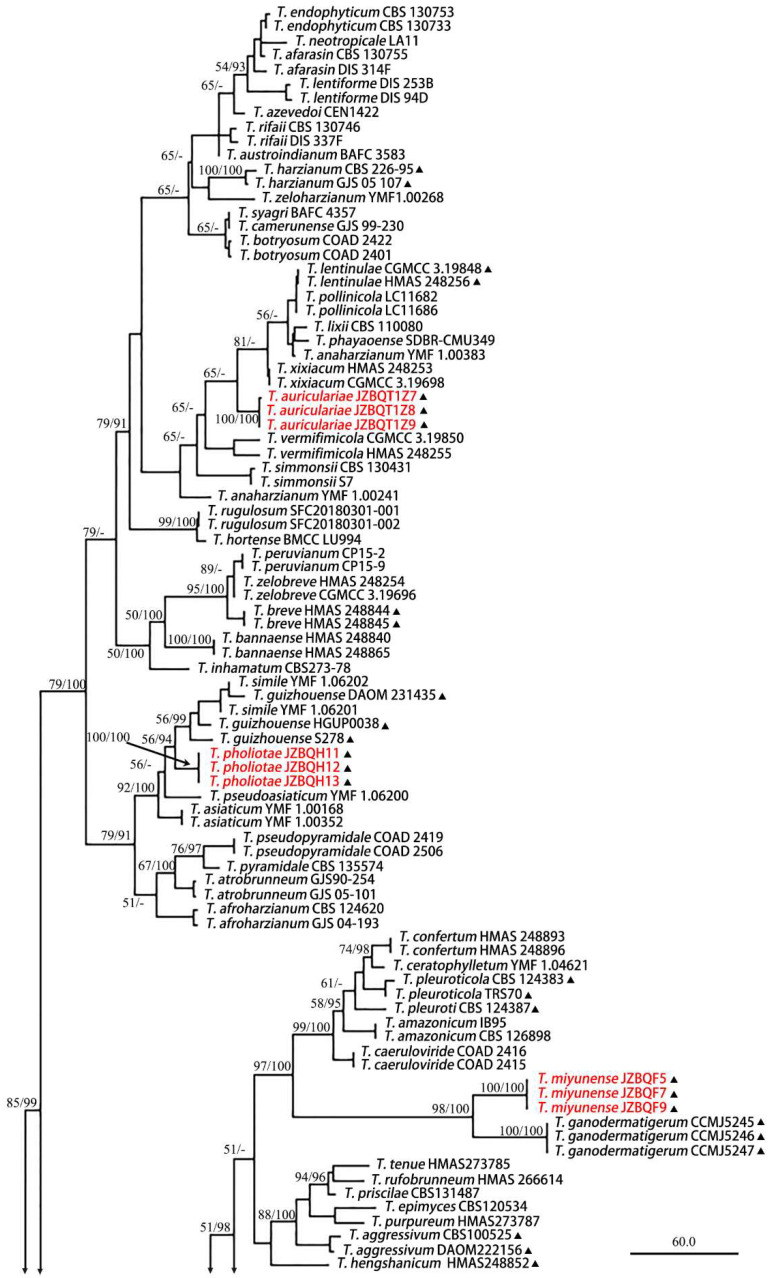

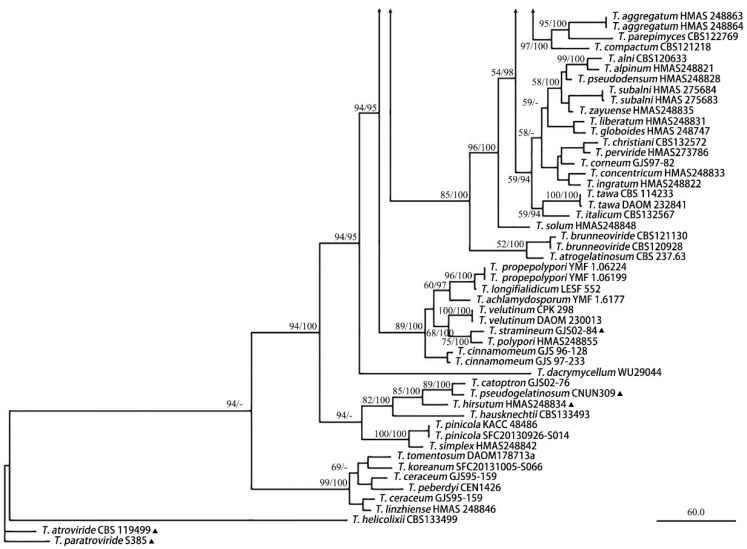
Maximum parsimony phylogram of the investigated *Trichoderma* species inferred from the combined sequences of *rpb2* and *tef1-α*. MPBP above 50% (left) and BIPP above 90% (right) are indicated at the nodes. New species proposed are indicated in red font. *Trichoderma* species isolated from substrate or fruiting bodies of edible fungi are marked with ▲.

The partition homogeneity test of *rpb2* and *tef1-α* sequences indicated that the individual partitions were generally congruent (*p* = 0.01). The combined *rpb2* and *tef1-α* dataset was subsequently used for phylogenetic analysis to determine the positions of the new species. In MP analysis, the dataset contained 140 taxa and 2307 characters, of which 1468 characters were constant, 150 variable characters were parsimony uninformative, and 689 were parsimony informative. Five most parsimonious trees with the same topology were generated, and one of them is shown in Figure 1 (tree length = 3091, CI = 0.3999, HI = 0.6001, RC = 0.3039, and RI = 0.7600). The BI tree topology was generally the same as that of the MP tree.

A total of 140 sequences representing 95 *Trichoderma* species, including our three new species, were used for constructing the phylogenetic tree, and *T. atroviride* and *T. paratroviride* were used as outgroups. Results showed that all the investigated *Trichoderma* species formed a strongly supported group (MPBP/BIPP = 100%/100%), which was generally congruent with the previous studies [24].

In the phylogenetic tree (Figure 1), *T. auriculariae*, *T. miyunense*, and *T. pholiotae* were newly added to the *T. harzianum* clade. *Trichoderma auriculariae* was distributed as a separate terminal branch (MPBP/BIPP = 100%/100%) among *T. vermifimicola* and *T. xixiacum*. *Trichoderma miyunense* was a sister of *T. ganodermatigerum* (MPBP/BIPP = 98%/100%). *Trichoderma pholiotae* formed a linage with *T. asiaticum*, *T. guizhouense*, *T. pseudoasiaticum*, and *T. simile* with high support value (MPBP/BIPP = 92%/100%), and our three strains of *T. pholiotae* were distributed as a highly supported separate terminal branch (MPBP/BIPP = 100%/100%) among *T. pseudoasiaticum* and *T. guizhouense*.

### 3.2. Taxonomy

*Trichoderma auriculariae* Z. J. Cao and W.T. Qin, sp. nov.

**Figure 2:**
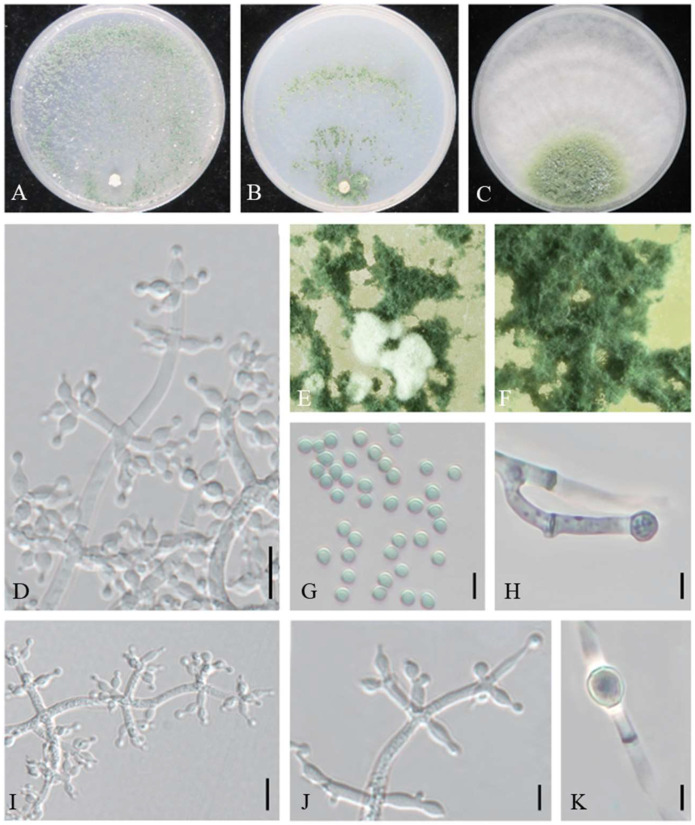
*Trichoderma auriculariae* (JZBQT1Z7). Cultures at 25 °C after 7 days on (**A**) CMD, (**B**) SNA, and (**C**) PDA; (**D**,**I**,**J**) conidiophores and phialides; (**E**,**F**) conidiation pustules on CMD after 7 days; (**G**) conidia; (**H**,**K**) chlamydospores. Scale bars: (**D**,**I**) = 10 µm, (**G**,**H**,**J**,**K**) = 5 µm.

MycoBank MB845141 (Figure 2).

Etymology: The specific epithet refers to the host from which the fungus was isolated.

Typification: China, Beijing, Tongzhou, from the contaminated substrates of *Auricularia heimuer*, 26 August 2021, W.T. Qin, Z.J. Cao, L. Gao, J. Li (ex-type strain JZBQT1Z7).

DNA barcodes: ITS = ON653396, *rpb2* = ON649949, *tef1*-*α* = ON649896.

On CMD after 72 h, colony radius 65–66 mm at 25 °C, 69–70 mm at 30 °C, and 8–10 mm at 35 °C. Colony hyaline and radial, not zonate. Aerial hyphae rare in colony center. A large number of white pustules formed after 2 days. Conidiation formed on aerial hyphae and in pustules, abundant, spreaded throughout the colony, then gradually turned green. No diffusing pigment noted.

On PDA after 72 h, colony radius 47–49 mm at 25 °C, 66–68 mm 30 °C, and 5–7 mm at 35 °C. Colony regularly circular, distinctly zonate. Aerial mycelium dense and radial, forming a dense, zonate, floccose mat. Conidial production noted after 2 days, starting around the original inoculum, effuse in aerial hyphae, more abundant along the original inoculum. No diffusing pigment noted, odor fruity.

On SNA after 72 h, colony radius 47–49 mm at 25 °C, 51–55 mm at 30 °C, and 5–7 mm at 35 °C. Colony hyaline, mycelium loose. Conidial production noted after 2 days, starting around the inoculum, effuse in the aerial hyphae, forming a few inconspicuous rings. Small pustules formed around the inoculum, first white, turning green after 3 days, with hairs protruding beyond the surface. No diffusing pigment.

Conidiophores pyramidal, with opposing branches borne on a conspicuously broad spindle, less solitary. The main axis and branches terminating in 3–5 cruciate to nearly verticillate disposed phialides. Hyphal septa clearly visible. Phialides ampulliform, sometime lageniform, 4.6–9.9 × (2.2–) 2.7–3.8 µm, l/w 1.4–3.5 (–4.4), 1.4–2.7 µm wide at the base (n = 50). Conidia green, globose or subglobose, sometimes ellipsoidal, smooth, 2.7–3.8 × 2.3–3.1 µm, l/w 1.0–1.3 (n = 50). Chlamydospores common, intercalary or terminal, variable in shape, ellipsoid, globose or oblong, 4.6–7.5 × 3.8–6.3 µm (n = 20).

Additional strains examined: China, Beijing, Tongzhou, from the contaminated substrates of *A. heimuer*, 26 August 2021, W.T. Qin, Y. Liu, S.X. Wang, JZBQT1Z8; *ibid*., JZBQT1Z9.

Notes: Phylogenetically, *T. auriculariae* formed a separate group (MPBP/BIPP = 100%/100%) in the Harzianum clade among *T. vermifimicola* and *T. xixiacum*. The *tef1-α* sequences between *T. auriculariae* and *T. vermifimicola* were very similar, but they shared 28 bp divergent among 1117 bp for *rpb2* sequences (97.49%). Phylogenetically, *T. auriculariae* shared a common ancestor with *T. xixiacum*, *T. vermifimicola*, and *T. simmonsi*. *Trichoderma auriculariae* shared typical characteristics of the Harzianum clade in pyramidal conidiophores comprising a long main axis, and 3–5 phialides in whorls arose at the tips of the branches. However *T. auriculariae* had longer phialides and grew much slower at 35 °C on PDA than *T. simmonsi* [5.2–6.5 mm, 25–55 mm] [25] and had larger conidia than that of *T. vermifimicola* [2.3–2.6 × 2.0–2.4 µm] and *T. xixiacum* [2.3–2.7 × 2.0–2.6] [24]. Meanwhile, chlamydospores were common in *T. auriculariae* (Table S1).

*Trichoderma miyunense* Z. J. Cao and W.T. Qin, sp. nov.

**Figure 3:**
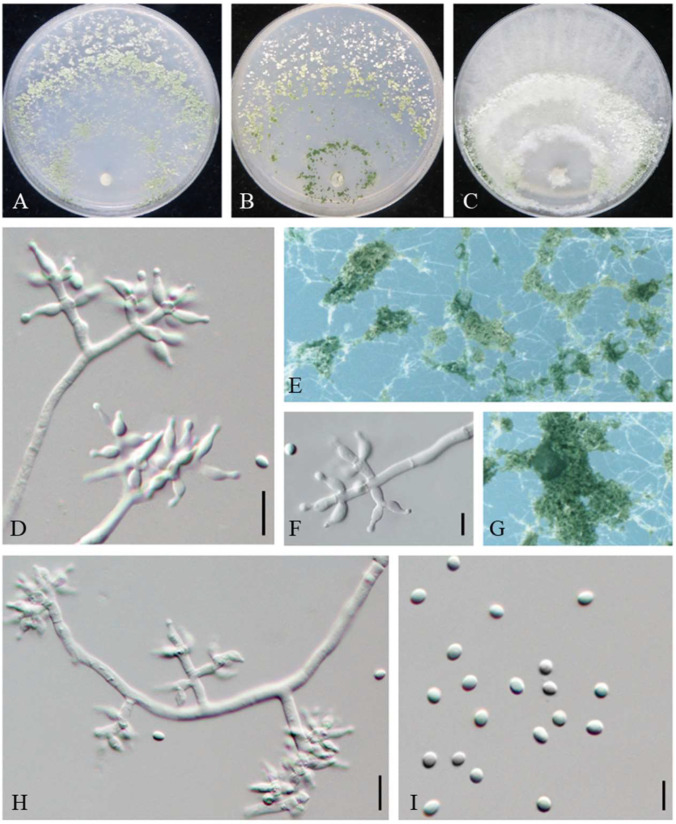
*Trichoderma miyunense* (JZBQF9). Cultures at 25 °C after 7 days on (**A**) CMD, (**B**) SNA, and (**C**) PDA; (**D**,**F**,**H**) conidiophores and phialides; (**E**,**G**) conidiation pustules on SNA after 7 days; (**I**) chlamydospores. Scale bars: (**D**,**H**) = 10 µm, (**F**,**I**) = 5 µm.

MycoBank MB845142 (Figure 3).

Etymology: The specific epithet refers to the type locality.

Typification: China, Beijing, Miyun, from the contaminated substrates of *Auricularia heimuer*, 9 September 2020, Y. Liu, W.T. Qin, S. Song (ex-type strain JZBQF9).

DNA barcodes: ITS = ON653404, *rpb2* = ON649970, *tef1*-*α* = ON649917.

On CMD after 72 h, colony radius 51–52 mm at 25 °C and 65–66 mm at 30 °C. No growth at 35 °C. Colony hyaline, weak, regularly circular, distinctly zonate. Conidiation first formed in white pustules on aerial hyphae, turned green after a few days. No diffusing pigment noted, odor slightly fruity.

On PDA after 72 h, colony radius 42–43 mm at 25 °C and 51–54 mm at 30 °C. No growth at 35 °C. Mycelium white, aerial along the edge, irregularly circular, less with sporulation. No diffusing pigment noted, odor slightly fruity.

On SNA after 72 h, colony radius 30–33 mm at 25 °C and 25–29 mm at 30 °C. No growth at 35 °C. Mycelium hyaline and smooth, dark green to light green pustules, irregular in shape, relatively abundant in the zonation regions, with the formation of 2–3 concentric rings. Aerial hyphae short and inconspicuous. No diffusing pigment, no distinct odor.

Conidiophores pyramidal, with a relatively obvious main axis, multiple branches unpaired, with the longest branches near the base of the main axis. Branches perpendicular to the main axis or at acute angles with the main axis, with septa conspicuous and producing barrel-shaped or cylindrical metulae. Phialides densely disposed at the terminal of branches, often formed in whorls of 2–4, variable in shape and size, ampulliform to lageniform, (5.2–) 5.6–9.7 (–10.3) × 1.9–3.2 (–3.7) µm, l/w 1.9–4.4, 1.0–2.1 (–2.6) wide at the base (n = 80). Conidia green, smooth, ellipsoid, sometimes globose to subglobose, 2.2–3.4 × (1.8–) 2–2.9 µm, l/w 1–1.3 (–1.4) (n = 80). Chlamydospores unobserved.

Additional strains examined: China, Beijing, Miyun, from the contaminated substrates of *Auricularia heimuer*, 9 September 2020, W.T. Qin, Y. Liu, S. Song, JZBQF5; *ibid.*, JZBQF7.

Notes: Phylogenetically, *T. miyunense* formed a sister group with *T. ganodermatigerum* (Figure 1). They shared 36 bp divergent among 1132 bp for *rpb2* sequences (96.82%) and 35 bp divergent among 1102 bp for *tef1*-*α* sequences (96.82%). Morphologically, compared to *T. miyunense*, *T. ceratophylletum* possessed shorter phialides (4.1–8.4 µm) and lesser l/w of phialides [(1.0–) 1.2–2.8 (–3.2) µm] [26], while *T. ganodermatigerum* had larger conidia [(3.4–) 3.6–4.8 (–5.3) × (2.9–) 3.2–4.3 (–4.6)], and the optimum temperature was 25 °C [27]. *T. miyunense* was distinctly different from *T. caeruloviride*, which possessed abundant chlamydospores on CMD after 4 days with no concentric rings present [28]. In contrast, *T. confertum* had slightly larger phialides [8.3–12.5 × 2.5–4.2 µm] [29], *T. amazonicum* had distinctly wider phialides [3.3–3.5 µm] and chlamydospore-like structures in the clusters, and *T. pleuroticola* featured diffuse brown pigment and yellow crystals on PDA [30] (Table S2).

*Trichoderma pholiotae* Z.J. Cao & W.T. Qin, sp. nov.

**Figure 4:**
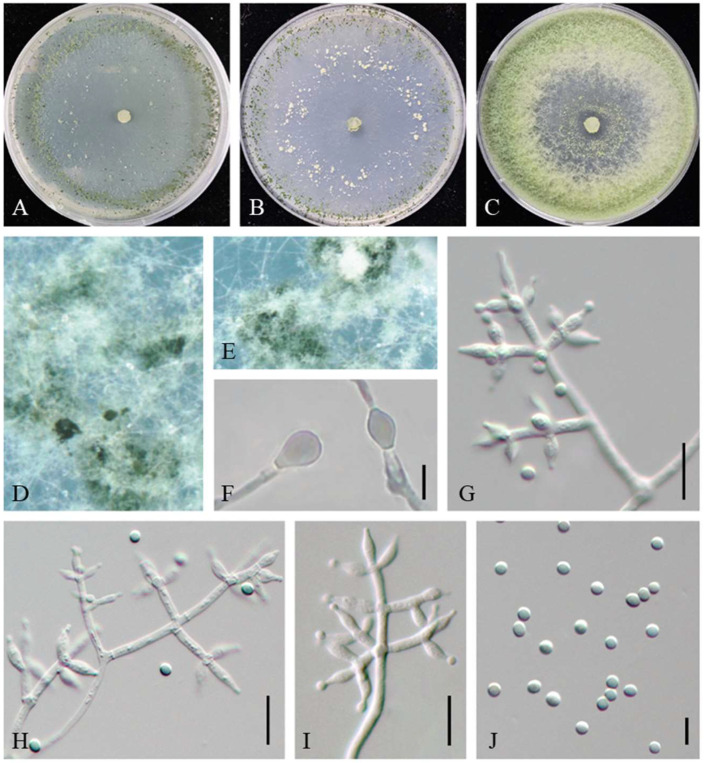
*Trichoderma pholiotae* (JZBQH12). Cultures at 25 °C after 7 days on (**A**) CMD, (**B**) SNA, and (**C**) PDA; (**D**,**E**) conidiation pustules on SNA after 7 days; (**F**) chlamydospores; (**G**–**I**) conidiophores and phialides; (**J**) conidia. Scale bars: (**F**,**J**) = 5 µm, (**G**–**I**) = 10 µm.

MycoBank MB845143 (Figure 4).

Etymology: The specific epithet refers to the host from which the fungus was isolated.

Typification: China, Beijing, Haidian, from the contaminated substrates of *Pholiota adipose*, 25 September 2020, W.T. Qin, Z.J. Cao, L. Gao, J. Li (ex-type strain JZBQH12).

DNA barcodes: ITS = ON653405, *rpb2* = ON649972, *tef1*-*α* = ON649919.

On CMD after 72 h, colony radius 71–72 mm at 25 °C, 73–74 mm at 30 °C, and 13–18 mm at 35 °C. Colonies hyaline, fan-shaped, tending to aggregate toward the distal parts of the colony. Aerial hyphae loose, sparse, radial. Conidiation effuse in aerial hyphae or in loosely disposed pustules. Pustules minute, irregular in shape, relatively abundant in the zonation regions, formed concentric rings around the outer ring, white at first, then gradually green. No diffusing pigment noted, odor slightly fruity.

On PDA after 72 h, colony radius 67–68 mm at 25 °C, 70–72 mm at 30 °C, and 8–10 mm at 35 °C. Colonies white in the center, with the zone around the central part of the colony forming a distinct circular and green part. Aerial hyphae distinctly radial, abundant, dense, floccose to cottony. Light diffusing yellow pigment, odor slightly fruity.

On SNA after 72 h, colony radius 49–50 mm at 25 °C, 54–55 mm at 30 °C, and 8–10 mm at 35 °C. Colonies translucent and round-like. Aerial hyphae short, radial distribution. Pustules abundant, irregular in shape, from white to green, with the formation of concentric rings. No diffusing pigment noted.

Conidiophores typically pyramidal with opposing branches, formed densely intricate reticulum, with one terminal whorl of generally 3–4 phialides and mostly paired side branches, less frequently solitary. Branches mostly perpendicular to the main axis with septa conspicuous. Phialides varied, borne in regular levels around the axis, some regular ampulliform or lageniform and others apex and inequilateral to curved, (4.1–) 4.9–10.9 (–11.6) × 2.4–4.2 (–5.0) µm, l/w 1.4–3.4 (–3.9), (1.3–) 1.4–3.1 (–3.4) µm wide at the base (n = 100). Conidia elliptic to subspheroidal, less globose, green, smooth, 2.6–3.8 (–4.2) × 2.4–3.3 (–3.5) µm, l/w 1–1.3 (n = 80). Chlamydospores common, intercalary or terminal, ellipsoid, globose, 5.0–7.4 (8.3) × (3.9–) 4.9–7.0 µm (n = 25).

Additional strains examined: China, Beijing, Haidian, from the contaminated substrates of *Pholiota adipose*, 25 September 2020, W.T. Qin, Z.J. Cao, L. Gao, J. Li, JZBQH11; *ibid.*, JZBQH13.

Notes: Phylogenetically, *T. pholiotae* formed a linage with *T. asiaticum*, *T. guizhouense*, *T. pseudoasiaticum*, and *T. simile* with high support value (MPBP/BIPP = 92%/100%), and our three strains of *T. pholiotae* were distributed as a highly supported separate terminal branch (MPBP/BIPP = 100%/100%) among *T. pseudoasiaticum* and *T. guizhouense* in the Harzianum clade. However, compared to *T. pholiotae*, *T. guizhouense* possessed thinner phialides [2.0–3.0 µm] and globose conidia [31]. *T. simile* had distinct lower optimum growth temperature (25 °C) in the three media, and *T. asiaticum* had shorter phialides [(3.0–) 4.0–6.0 (–7.0) µm] [12]. In addition, *T. pholiotae* and *T. pseudoasiaticum* could be distinguished by the branching pattern, with *T. pholiotae* being pyramidal and *T. pseudoasiaticum* being verticillium-like (Table S3).

## 4. Discussion

During exploration of contaminated substrates of edible fungi in North China, 39 strains representing 10 *Trichoderma* species were isolated from four kinds of edible fungi and examined, and three new species were recognized based on integrated studies of phenotypic and molecular data (Table S1). To explore their taxonomic positions, a phylogenetic tree containing all species of the Harzianum clade was constructed based on analyses of the combined sequences of *rpb2* and *tef1*-*α*. The three new species were well located in the Harzianum clade with separate terminal branches and were clearly distinguishable from any of the existing species. The results of this study have a number of practical implications to identify and diagnose *Trichoderma* species contaminating edible fungi. This work provides useful information on the epidemiological and geographical distribution of *Trichoderma*, which will help in the development of targeted interventions aimed at comprehensive management and control of green mold contamination of edible fungi.

With further study of *Trichoderma* classification, researchers have reached a consensus that accurate identification of *Trichoderma* species cannot depend only on the morphological identification as sometimes there is high ambiguity in the morphological features of *Trichoderma* spp. [32,33]. *Trichoderma* spp. isolated from the fruiting bodies or substrates of edible fungi is usually anamorph with high morphological similarity with many species, which is not conducive to identification. With DNA-based techniques gradually perfected and widely used, the integrative (polyphasic) taxonomy approach for species delimitation is recommended, including the combination of genealogy and multiparametric phenotypes [34,35], especially for examining the presence of species complexes and cryptic species [31]. Therefore, we hypothesized that *T. harzianum*, which was originally identified by ITS sequence and morphology in previous studies, probably belonged to the *T. harzianum* complex. However, the present study showed that the complex still contained many taxa, indicating that the previous identification was not accurate. Furthermore, it is also difficult to identify species of the Harzianum clade according to exclusive *tef1*-*α* or *rpb2* sequence data [24,25]. Therefore, the combination of *tef1*-*α* and *rpb2* sequences for phylogenetic analysis is highly recommended to identify species in the Harzianum clade.

Taxonomy of *Trichoderma* dates back to the late 18th century [36], and some of them cause economic losses in commercial mushroom farms [37]. Over more than a century, successive findings have brought the number of known species of the genus to over 441 [1,23,38]. *Trichoderma* species are located throughout the world, and more than 30 of them are mushroom inhabiting (Figure 1, Table 2). They are isolated from the substrate or fruiting bodies of *Agaricus bisporus*, *Lentinula edodes*, *Pleurotus ostreatus*, *Ganoderma lingzhi*, etc. and are mainly located in the Harzianum, Longibrachiatum, and Viride clades [39]. There may still be many unknown *Trichoderma* species associated with the growth of edible fungi and their related living environment. The phylogenetical difference between *Trichoderma* spp. on edible fungi substrates and from other sources deserves further analysis.

Analysis of the biological characteristics of *Trichoderma* species from contaminated substrates showed that the optimum growth temperature of many *Trichoderma* species was generally around 30 °C, which was consistent with the phenomenon that contamination of *Trichoderma* on edible fungi is more likely to occur at high temperatures. Therefore, reasonable control the growth environment temperature of edible fungi may be a reasonable approach to prevent or delay the outbreak of *Trichoderma* contamination during production. More broadly, research is also needed to analyze the mechanism of occurrence of *Trichoderma* spp. contamination, such as the correlation between contamination occurrence and the growth environment of edible fungi.

With the increased number of species joining the Harzianum clade, understanding of *Trichoderma* spp. will become more sophisticated and intelligible, and reasonable species concepts will be firmly established. Accumulated knowledge of *Trichoderma*, especially the Harzianum clade, will provide useful information for sufficient utilization of resources and for the prevention of contamination of edible fungi.

## 5. Conclusions

In this study, 39 strains belonging to 10 *Trichoderma* species isolated from four kinds of edible fungi in North China were obtained, and three novel species belonging to the Harzianum clade were isolated from the contaminated substrates of *Auricularia heimuer* and *Pholiota adipose*. More than 30 mushroom-inhabiting *Trichoderma* species throughout the world mainly located in the Harzianum, Longibrachiatum, and Viride clades were indicated. This study enrich the biodiversity of *Trichoderma* and provide important support for systematic development of the Harzianum clade.

## Figures and Tables

**Table 1:** Materials including strain numbers and GenBank accessions of sequences used for phylogenetic analyses.

Species	Voucher	GenBank Accession Number	
		*rpb2*	*tef1*-*α*
*T. achlamydosporum*	YMF 1.6177	MT052180	MT070156
*T. afarasin*	CBS 130755	–	AF348093
*T. afarasin*	DIS 314F	FJ442778	FJ463400
*T. afroharzianum*	CBS 124620 ^ET^	FJ442691	FJ463301
*T. afroharzianum*	GJS 04-193	FJ442709	FJ463298
*T. aggregatum*	HMAS 248863	KY688001	KY688062
*T. aggregatum*	HMAS 248864	KY688002	KY688063
*T. aggressivum*	CBS 100525	AF545541	AF348095
*T. aggressivum*	DAOM 222156 ^ET^	FJ442752	AF348098
*T. alni*	CBS 120633 ^ET^	EU498349	EU498312
*T. alpinum*	HMAS 248821 ^T^	KY687958	KY688012
*T. amazonicum*	IB95	HM142368	HM142377
*T. amazonicum*	CBS126898 ^ET^	HM142367	HM142376
*T. anaharzianum*	YMF 1.00241	MH262577	MH236493
*T. anaharzianum*	YMF 1.00383 ^T^	MH158995	MH183182
*T. asiaticum*	YMF 1.00168	MH262575	MH236492
*T. asiaticum*	YMF 1.00352 ^T^	MH158994	MH183183
*T. atrobrunneum*	GJS90-254	FJ442735	AF443943
*T. atrobrunneum*	GJS 05-101	FJ442745	FJ463392
*T. atrogelatinosum*	CBS 237.63 ^ET^	KJ842201	–
*T. atroviride*	CBS 119499	FJ860518	FJ860611
*T. auriculariae*	JZBQT1Z7 ^T^	**ON649949**	**ON649896**
*T. auriculariae*	JZBQT1Z8	**ON649950**	**ON649897**
*T. auriculariae*	JZBQT1Z9	**ON649951**	**ON649898**
*T. austroindianum*	BAFC 3583	–	MH352421
*T. azevedoi*	CEN1422	MK696821	MK696660
*T. bannaense*	HMAS 248840 ^T^	KY687979	KY688037
*T. bannaense*	HMAS 248865	KY688003	KY688038
*T. botryosum*	COAD 2422	MK044212	MK044119
*T. botryosum*	COAD 2401	MK044181	MK044088
*T. breve*	HMAS 248844 ^T^	KY687983	KY688045
*T. breve*	HMAS 248845	KY687984	KY688046
*T. brunneoviride*	CBS 121130	EU498357	EU498316
*T. brunneoviride*	CBS 120928	EU498358	EU498318
*T. caeruloviride*	COAD 2416	MK044201	MK044108
*T. caeruloviride*	COAD 2415	MK044202	MK044109
*T. camerunense*	GJS 99-230	–	AF348107
*T. catoptron*	GJS 02-76 ^T^	AY391900	AY737726
*T. ceraceum*	GJS 95-159	AF545508	AY937437
*T. ceratophylletum*	YMF 1.04621 ^T^	MK327580	MK327579
*T. cerinum*	DAOM 230012	KJ842184	KJ871242
*T. christiani*	CBS 132572 ^ET^	KJ665244	KJ665439
*T. cinnamomeum*	GJS 96-128	AY391916	AY391977
*T. cinnamomeum*	GJS 97-233	AY391919	AY391978
*T. compactum*	CBS 121218	KF134789	KF134798
*T. concentricum*	HMAS 248833	KY687971	KY688027
*T. confertum*	HMAS 248893	MF371203	MF371218
*T. confertum*	HMAS 248896	MF371205	MF371220
*T. corneum*	GJS 97-82 ^ET^	KJ665252	KJ665455
*T. dacrymycellum*	WU29044	FJ860533	FJ860633
*T. endophyticum*	CBS 130753	FJ442722	FJ463326
*T. endophyticum*	CBS 130733	FJ442690	FJ463330
*T. epimyces*	CBS120534 ^ET^	EU498360	EU498320
*T. ganodermatigerum*	CCMJ5245 ^T^	ON567189	ON567195
*T. ganodermatigerum*	CCMJ5246	ON567190	ON567196
*T. ganodermatigerum*	CCMJ5247	ON567191	ON567197
*T. globoides*	HMAS 248747	KX026963	KX026955
*T. guizhouense*	HGUP0038 ^T^	JQ901400	JN215484
*T. guizhouense*	S278	KF134791	KF134799
*T. guizhouense*	DAOM 231435	–	EF191321
*T. harzianum*	CBS 226-95	AF545549	AF348101
*T. harzianum*	GJS 05 107	FJ442708	FJ463329
*T. hausknechtii*	CBS 133493	KJ665276	KJ665515
*T. helicolixii*	CBS 133499	KJ665278	KJ665517
*T. hengshanicum*	HMAS 248852 ^T^	KY687991	KY688054
*T. hirsutum*	HMAS 248834 ^T^	KY687972	KY688029
*T. hortense*	BMCC LU994	–	KJ871185
*T. ingratum*	HMAS 248822	KY687973	KY688018
*T. inhamatum*	CBS 273-78	FJ442725	AF348099
*T. italicum*	CBS 132567	KJ665282	KJ665525
*T. koreanum*	SFC20131005-S066	MH025988	MH025979
*T. lentiforme*	DIS 253B	FJ442756	FJ851875
*T. lentiforme*	DIS 94D	FJ442749	FJ463379
*T. lentinulae*	HMAS 248256	MN605867	MN605878
*T. lentinulae*	CGMCC 3.19848	MN605868	MN605879
*T. liberatum*	HMAS 248831 ^T^	KY687969	KY688025
*T. linzhiense*	HMAS 248846 ^T^	KY687985	KY688047
*T. lixii*	CBS 110080	KJ665290	FJ716622
*T. longifialidicum*	LESF 552	KT278955	KT279020
*T. miyunense*	JZBQF5	**ON649968**	**ON649915**
*T. miyunense*	JZBQF7 ^T^	**ON649969**	**ON649916**
*T. miyunense*	JZBQF9	**ON649970**	**ON649917**
*T. neotropicale*	LA11 ^ET^	–	HQ022771
*T. paratroviride*	S385	KJ665321	KJ665627
*T. parepimyces*	CBS 122769 ^ET^	FJ860562	FJ860664
*T. peberdyi*	CEN1426	MK696825	MK696664
*T. peruvianum*	CP15-2	MW480153	MW480145
*T. peruvianum*	CP15-9	MW480154	MW480146
*T. perviride*	HMAS 273786	KX026962	KX026954
*T. phayaoense*	SDBR-CMU349	MW002074	MW002073
*T. pholiotae*	JZBQH11	**ON649971**	**ON649918**
*T. pholiotae*	JZBQH12 ^T^	**ON649972**	**ON649919**
*T. pholiotae*	JZBQH13	**ON649973**	**ON649920**
*T. pinicola*	KACC 48486 ^ET^	MH025993	MH025981
*T. pinicola*	SFC20130926-S014	MH025991	MH025978
*T. pleuroti*	CBS 124387 ^ET^	HM142372	HM142382
*T. pleuroticola*	CBS 124383 ^ET^	HM142371	HM142381
*T. pleuroticola*	TRS70 ^ET^	KP009172	KP008951
*T. pollinicola*	LC11682 = LF1542 ^ET^	MF939604	MF939619
*T. pollinicola*	LC11686 = LF2050	MF939605	MF939620
*T. polypori*	HMAS 248855 ^T^	KY687994	KY688058
*T. priscilae*	CBS 131487 ^ET^	KJ665333	KJ665691
*T. propepolypori*	YMF 1.06224 ^T^	MT052181	MT070158
*T. propepolypori*	YMF 1.06199	MT052182	MT070157
*T. pseudoasiaticum*	YMF 1.06200 ^T^	MT052183	MT070155
*T. pseudodensum*	HMAS 248828 ^T^	KY687967	KY688023
*T. pseudogelatinosum*	CNUN309 ^ET^	HM920173	HM920202
*T. pseudopyramidale*	COAD 2419	MK044206	MK044113
*T. pseudopyramidale*	COAD 2506	MK044207	MK044114
*T. purpureum*	HMAS 273787 ^T^	KX026961	KX026953
*T. pyramidale*	CBS 135574 ^ET^	KJ665334	KJ665699
*T. rifaii*	CBS 130746	–	FJ463324
*T. rifaii*	DIS 337F	FJ442720	FJ463321
*T. rufobrunneum*	HMAS 266614 ^T^	KF730010	KF729989
*T. rugulosum*	SFC20180301-001 ^T^	MH025986	MH025984
*T. rugulosum*	SFC20180301-002	MH025987	MH025985
*T. simile*	YMF 1.06201 ^T^	MT052184	MT070154
*T. simile*	YMF 1.06202	MT052185	MT070153
*T. simmonsii*	CBS 130431	FJ442757	AF443935
*T. simmonsii*	S7	KJ665337	KJ665719
*T. simplex*	HMAS 248842 ^T^	KY687981	KY688041
*T. solum*	HMAS 248848 ^T^	KY687987	KY688050
*T. stramineum*	GJS 02-84	AY391945	AY391999
*T. subalni*	HMAS 275683	MH612371	MH612377
*T. subalni*	HMAS 275684	MH612370	MH612376
*T. syagri*	BAFC 4357	–	MG822711
*T. tawa*	CBS 114233 ^ET^	AY391956	FJ463313
*T. tawa*	DAOM 232841	KJ842187	EU279972
*T. tenue*	HMAS 273785 ^ET^	KX026960	KX026952
*T. tomentosum*	DAOM 178713a	AF545557	AY750882
*T. velutinum*	CPK 298	KF134794	KJ665769
*T. velutinum*	DAOM 230013 ^ET^	JN133569	AY937415
*T. vermifimicola*	CGMCC 3.19850	MN605870	MN605881
*T. vermifimicola*	HMAS 248255	MN605871	MN605882
*T. xixiacum*	HMAS 248253 ^T^	MN605874	MN605885
*T. xixiacum*	CGMCC 3.19698	MN605875	MN605886
*T. zayuense*	HMAS 248835 ^T^	KY687974	KY688031
*T. zelobreve*	HMAS 248254 ^T^	MN605872	MN605883
*T. zelobreve*	CGMCC 3.19696	MN605873	MN605884
*T. zeloharzianum*	YMF 1.00268	MH158996	MH183181

Numbers in bold indicate newly submitted sequences in this study. ^T^: type strains. ^ET^: ex-type strains.

**Table 2:** *Trichoderma* spp. associated with the contaminated substrates of edible fungi.

Species	Cultivated Mushroom	Reference
*T. aggressivum*	*Agaricus bisporus*	[40,41]
*T. asperellum*	*A. bisporus*	[9,42]
*T. atroviride*	*L. edodes*, *Pleurotus ostreatus*, *A. bisporus*, *Ganoderma lingzhi*	[8,9,43]
*T. aureoviride*	*Auricularia heimuer*, *Flammulina filiformis*, *L. edodes*	[44]
*T. breve*	*L. edodes*	[45]
*T. capillare*	*Agaricus* sp.	[46]
*T. citrinviride*	*L. edodes*, *P. ostreatus*	[43,47]
*T. deliquescens*	*L. edodes*	[11]
*T. ganodermatigerum*	*G. sichuanense*	[27]
*T. ghanense*	*A. bisporus*	[9]
*T. guizhouense*	*P. ostreatus*	[48]
*T. hamatum*	*A. bisporus*	[49]
*T. harzianum*	*L. edodes*, *A. bisporus*, *P. ostreatus*, *Agrocybe aegerita*	[43,50]
*T. hengshanicum*	*G. lingzhi*	[51]
*T. hirsutum*	*L. edodes*	[45]
*T. koningii*	*P. ostreatus*, *A. bisporus*	[37,40]
*T. koningiopsis*	*Dictyophora rubrovolvata*, *P. eryngii*	[52,53]
*T. lentinulae*	*L. edodes*	[24]
*T. longibrachiatum*	*L. edodes*, *P. ostreatus*, *A. aegerita*	[9,43,50]
*T. oblongisporum*	*L. edodes*	[54]
*T. patella*	*P. ostreatus*	[55]
*T. pleuroti*	*P. ostreatus*	[56]
*T. pleuroticola*	*P. ostreatus*, *L. edodes*, *G. lingzhi*	[50,54,56]
*T. polysporum*	*L. edodes*	[57]
*T. pseudogelatinosum*	*L. edodes*	[58]
*T. pseudokoningii*	*P. ostreatus*	[37]
*T. pseudolacteum*	*L. edodes*	[59]
*T. pseudostramineum*	*L. edodes*	[58]
*T. reesei*	*P. ostreatus*	[60]
*T. stramineum*	*L. edodes*	[57]
*T. stromaticum*	*A. bisporus*	[49]
*T. virens*	*P. ostreatus*, *A. bisporus*	[37,40]
*T. viride*	*L. edodes*	[54]

## Data Availability

Not applicable.

## Supplementary Materials

The following supporting information can be downloaded at: https://www.mdpi.com/article/10.3390/jof8111154/s1. Table S1: Strain information and their accession numbers. Table S2: Comparison of the morphological characteristics of *Trichoderma auriculariae* and its relatives. Table S3: Comparison of the morphological characteristics of *Trichoderma miyunense* and its relatives. Table S4: Comparison of the morphological characteristics of *Trichoderma pholiotae* and its relatives. Table S5: The growth rate of three new species in this study incubated at different temperatures and media.

## References

[1] Cai F., Druzhinina I.S. (2021). In honor of John Bissett: Authoritative guidelines on molecular identification of *Trichoderma*. Fungal Divers..

[2] Druzhinina I.S., Seidl-Seiboth V., Herrera-Estrella A., Horwitz B.A., Kenerley C.M., Monte E., Mukherjee P.K., Zeilinger S., Grigoriev I.V., Kubicek C.P. (2011). *Trichoderma*: The genomics of opportunistic success. Nat. Rev. Microbiol..

[3] Nuangmek W., Aiduang W., Kumla J., Lumyong S., Suwannarach N. (2021). Evaluation of a newly identified endophytic fungus, *Trichoderma phayaoense* for plant growth promotion and biological control of gummy stem blight and wilt of muskmelon. Front. Microbiol..

[4] Carillo P., Woo S.L., Comite E., El-Nakhel C., Vinale F. (2020). Application of *Trichoderma harzianum*, 6-pentyl-α-pyrone and plant biopolymer formulations modulate plant metabolism and fruit quality of plum tomatoes. Plants.

[5] Wei H., Wu M., Fan A., Su H. (2021). Recombinant protein production in the filamentous fungus *Trichoderma*. Chin. J. Chem. Eng..

[6] Gupta S., Smith P., Boughton B., Twt R., Sha N., Roessner U. (2021). Inoculation of barley with *Trichoderma harzianum* T-22 modifies lipids and metabolites to improve salt tolerance. J. Exp. Bot..

[7] Samuel G.J., Dodd S.L., Gams W., Castlebury L.A., Petrini O. (2002). *Trichoderma* species associated with the green mold epidemic of commercially grown *Agaricus bisporus*. Mycologia.

[8] Yan Y., Zhang C., Moodley O., Zhang L., Xu J. (2019). Green mold caused by *Trichoderma atroviride* on the lingzhi medicinal mushroom, *Ganoderma lingzhi* (Agaricomycetes). Int. J. Med. Mushrooms.

[9] Hatvani L., Antal Z., Manczinger L., Szekeres A., Druzhinina I.S., Kubicek C.P., Nagy A., Nagy E., Vagvolgyi C., Kredics L. (2007). Green mold diseases of *Agaricus* and *Pleurotus* spp. are caused by related but phylogenetically different *Trichoderma* species. Phytopathology.

[10] Komon-Zelazowska M., Bissett J., Zafari D., Hatvani L., Manczinger L., Woo S., Lorito M., Kredics L., Kubicek C.P., Druzhinina I.S. (2007). Genetically closely related but phenotypically divergent *Trichoderma* species cause green mold disease in oyster mushroom farms worldwide. Appl. Environ. Microb..

[11] Kim J.Y., Yun Y.H., Hyun M.W., Kim M.H., Kim S.H. (2010). Identification and characterization of *Gliocladium viride* isolated from mushroom fly infested oak log beds used for shiitake cultivation. Mycobiology.

[12] Zheng H., Qiao M., Lv Y.F., Du X., Zhang K.Q., Yu Z.F. (2021). New species of *Trichoderma* isolated as endophytes and saprobes from southwest China. J. Fungi.

[13] Ignazio C., Linda M.K. (1999). A method for designing primer sets for speciation studies in filamentous ascomycetes. Mycologia.

[14] Jaklitsch W.M., Komon M., Kubicek C.P., Druzhinina I.S. (2005). *Hypocrea voglmayrii* sp. nov. from the Austrian Alps represents a new phylogenetic clade in *Hypocrea*/*Trichoderma*. Mycologia.

[15] Liu Y.J., Whelen S., Hall B.D. (1999). Phylogenetic relationships among ascomycetes: Evidence from an RNA polymerse II subunit. Mol. Biol. Evol..

[16] Zhu Z.X., Zhuang W.Y. (2015). *Trichoderma* (*Hypocrea*) species with green ascospores from China. Persoonia.

[17] Dou K., Lu Z., Wu Q., Ni M., Yu C., Wang M., Li Y., Wang X., Xie H., Chen J. (2020). MIST: A multilocus identification system for *Trichoderma*. Appl. Environ. Microb..

[18] Hall T.A. (1999). BioEdit: A user-friendly biological sequence alignment editor and analysis program for windows 95/98/NT. Nucleic Acids Symp..

[19] Higgins D.G., Jeanmougin F., Gibson T.J., Plewniak F., Thompson J.D. (1997). The Clustal X windows interface: Flexible strategies for multiple sequence alignment aided by quality analysis tools. Nucleic Acids Symp..

[20] Swofford D.L. (2002). PAUP*. Phylogenetic Analysis Using Parsimony (*and Other Methods).

[21] Ronquist F., Huelsenbeck J.P. (2003). MrBayes 3: Bayesian phylogenetic inference under mixed models. Bioinformatics.

[22] Nylander J.A.A. (2004). MrModeltest v2. Program Distributed by the Author. http://paup.csit.fsu.edu.

[23] Rambaut A. (2016). FigTree. Tree Figure Drawing Tool, v. 1.4.3. http://tree.bio.ed.ac.uk/.

[24] Gu X., Wang R., Sun Q., Wu B., Sun J.-Z. (2020). Four new species of *Trichoderma* in the Harzianum clade from northern China. Mycokeys.

[25] Chaverri P., Branco-Rocha F., Jaklitsch W., Gazis R., Degenkolb T., Samuels G.J. (2015). Systematics of the *Trichoderma harzianum* species complex and the re-identification of commercial biocontrol strains. Mycologia.

[26] Yuan H.S., Lu X., Dai Y.C., Hyde K.V.D., Kan Y.H., Kusan I., He S.H., Liu N.G., Sarma V.V., Zhao C.L. (2020). Fungal diversity notes 1277–1386: Taxonomic and phylogenetic contributions to fungal taxa. Fungal Divers..

[27] An X.Y., Cheng G.H., Gao H.X., Li D., Li Y. (2022). Phylogenetic analysis of *trichoderma* species associated with green mold disease on mushrooms and two new pathogens on *Ganoderma sichuanense*. J. Fungi.

[28] Rodriguez M.D.H., Evans H.C., De Abreu L.M., De Macedo D.M., Ndacnou M.K., Bekele K.B., Barreto R.W. (2021). New species and records of *Trichoderma* isolated as mycoparasites and endophytes from cultivated and wild coffee in Africa. Sci. Rep..

[29] Chen K., Zhuang W.Y. (2017). Seven new species of *Trichoderma* from soil in China. Mycosystema.

[30] Chaverri P., Gazis R.O., Samuels G.J. (2011). *Trichoderma amazonicum*, a new endophytic species on *Hevea brasiliensis* and *H. guianensis* from the Amazon basin. Mycologia.

[31] Li Q.R., Tan P., Jiang Y.L., Hyde K.D., Mckenzie E.H.C., Bahkali A.H., Kang J.C., Wang Y. (2013). A novel *Trichoderma* species isolated from soil in Guizhou, *T. guizhouense*. Mycol. Prog..

[32] Chaverri P., Samuels G.J. (2003). *Hypocrea*/*Trichoderma* (Ascomycota, Hypocreales, Hypocreaceae): Species with green ascospores. Stud. Mycol..

[33] Jaklitsch W.M., Samuels G.J., Dodd S.L., Lu B.S., Druzhinina I.S. (2006). *Hypocrea rufa*/*Trichoderma viride*: A reassessment, and description of five closely related species with and without warted conidia. Stud. Mycol..

[34] Luecking R., Aime M.C., Robbertse B., Miller A.N., Ariyawansa H.A., Aoki T., Cardinali G., Crous P.W., Druzhinina I.S., Geiser D.M. (2020). Unambiguous identification of fungi: Where do we stand and how accurate and precise is fungal DNA barcoding?. IMA Fungus.

[35] Druzhinina I., Kubicek C.P. (2005). Species concepts and biodiversity in *Trichoderma* and *Hypocrea*: From aggregate species to species clusters?. J. Zhejiang Univ. Sci. B.

[36] Persoon C.H. (1794). *Neurospora* genetic nomenclature. Romers Neues Mag. Bot..

[37] Jhune C.S., Leem H.T., Park H.S., Lee C.J., Weon H.Y., Seok S.J., Yoo K.H., Sung G.H. (2014). Identification of oyster mushroom green mold pathogen that causes and pathological characteristics. J. Mushrooms.

[38] Barrera V.A., Iannone L., Romero A.I., Chaverri P. (2021). Expanding the *Trichoderma harzianum* species complex: Three new species from Argentine natural and cultivated ecosystems. Mycologia.

[39] Allaga H., Zhumakayev A., Buchner R., Kocsube S., Szucs A., Vagvolgyi C., Kredics L., Hatvani L. (2021). Members of the *Trichoderma harzianum* species complex with mushroom pathogenic potential. Agronomy.

[40] Kosanovic D., Potocnik I., Duduk B., Vukojevic J., Stajic M., Rekanovic E., Milijasevic-Marcic S. (2013). *Trichoderma* species on *Agaricus bisporus* farms in Serbia and their biocontrol. Ann. Appl. Biol..

[41] Marik T., Urban P., Tyagi C., Szekeres A., Leitgeb B., Vagvolgyi M., Manczinger L., Druzhinina I.S., Vagvolgyi C., Kredics L. (2017). Diversity profile and dynamics of peptaibols produced by green mould *Trichoderma* species in interactions with their hosts *Agaricus bisporus* and *Pleurotus ostreatus*. Chem. Biodivers..

[42] Wu X.J., Hu F.P., He H.Z., Xie B.G. (2008). Identification of *Trichoderma* species associated with cultivated edible fungi. J. Agric. Biotechnol..

[43] Kim C.S., Park M.S., Kim S.C., Maekawa N., Yu S.H. (2012). Identification of *Trichoderma*, a competitor of shiitake mushroom (*Lentinula edodes*), and competition between *Lentinula edodes* and *Trichoderma* species in Korea. Plant Pathol. J..

[44] Cui L.H. (2017). Isolation, Identification and Diversity Analysis of the Contaminating Fungi from the Edible Mushroom-Growing Synthetic Wood Logs. Master’s Thesis.

[45] Wang Y. (2021). Identification of the Pathogen of *Lentinus edodes* Sticks Rot and Preliminary Study on Its Occurrence. Master’s Thesis.

[46] Samuels G.J., Ismaiel A., Mulaw T.B., Szakacs G., Druzhinina I.S., Kubicek C.P., Jaklitsch W.M. (2012). The Longibrachiatum clade of *Trichoderma*: A revision with new species. Fungal Divers..

[47] Park M.S., Seo G.S., Bae K.S., Yu S.H. (2005). Characterization of *Trichoderma* spp. associated with green mold of oyster mushroom by PCR-RFLP and sequence analysis of ITS regions of rDNA. Plant Pathol. J..

[48] Innocenti G., Montanari M., Righini H., Roberti R. (2019). *Trichoderma* species associated with green mould disease of *Pleurotus ostreatus* and their sensitivity to prochloraz. Plant Pathol..

[49] Song X.X., Wang Q., Jun J.X., Zhang J.J., Chen H., Chen M.J., Huang J.C., Xie B.Q. (2019). Study on accurate identification of four *Trichoderma* diseases in *Agaricus bisporus* in factory cultivation. Edible Fungi.

[50] Choi I.Y., Choi J.N., Hyu L.W., Sharma P.K. (2010). Isolation and identification of mushroom pathogens from *Agrocybe aegerita*. Mycobiology.

[51] Cai M., Idrees M., Zhou Y., Zhang C., Xu J. (2020). First report of green mold disease caused by *Trichoderma hengshanicum* on *Ganoderma lingzhi*. Mycobiology.

[52] Chen X., Zhou X., Zhao J., Tang X., Pasquali M., Migheli Q., Berg G., Cernava T. (2021). Occurrence of green mold disease on *Dictyophora rubrovolvata* caused by *Trichoderma koningiopsis*. J. Plant Pathol..

[53] Kim S.W., Kim S., Lee H.J., Park J.W., Ro H.S. (2013). Isolation of fungal pathogens to an edible mushroom, *Pleurotus eryngii*, and development of specific ITS primers. Mycobiology.

[54] Wang G., Cao X., Ma X., Guo M., Liu C., Yan L., Bian Y. (2016). Diversity and effect of *Trichoderma* spp. associated with green mold disease on *Lentinula edodes* in China. Microbiologyopen.

[55] Hua R., Li J.Y., Liu S.X., Luo X.K., Wang X.Y., Liu C.L., Zhang L.Y., Sun D.F. (2021). Investigation on common dieases of *Pleurotus ostreatus* and identification of pathogens. Edible Fungi China.

[56] Park M.S., Bae K.S., Yu S.H. (2006). Two new species of *Trichoderma* associated with green mold of oyster mushroom cultivation in Korea. Mycobiology.

[57] Miyazaki K., Tsuchiya Y., Okuda T. (2009). Specific PCR assays for the detection of *Trichoderma harzianum* causing green mold disease during mushroom cultivation. Mycoscience.

[58] Kim C.S., Yu S.H., Nakagiri A., Shirouzu T., Sotome K., Kim S.C., Maekawa N. (2012). Re-evaluation of *Hypocrea pseudogelatinosa* and *H. pseudostraminea* isolated from shiitake mushroom (*Lentinula edodes*) cultivation in Korea and Japan. Plant Pathol. J..

[59] Kim C.S., Shirouzu T., Nakagiri A., Sotome K., Maekawa N. (2013). *Trichoderma eijii* and *T. pseudolacteum*, two new species from Japan. Mycol. Prog..

[60] Lan B.M. (2022). Isolation and identification of *Trichoderma* on oyster mushroom in Quanzhou. Chin. J. Trop. Agric..

