# Supplementary material for: Three New Trichoderma Species in Harzianum Clade Associated with the Contaminated Substrates of Edible Fungi
Source: J Fungi (Basel). 2022 Oct 31;8(11):1154. doi: 10.3390/jof8111154 (PMC9696741; doi:10.3390/jof8111154)
Supplement: Supplementary file 1 [file jof-08-01154-s001.zip › jof-1936840-supplementary.pdf]

Supplementary Table S1. Strain information and their accession numbers.

Isolation	Species	Host	Source	Date	Accession	
					rpb2	*tef1-α*
JZBQF5	*T. miyunense*	*Auricularia heimuer*	Beijing, Miyun	2020.9.9	ON649968	ON649915
JZBQF7	*T. miyunense*	*Auricularia heimuer*	Beijing, Miyun	2020.9.9	ON649969	ON649916
JZBQF9	*T. miyunense*	*Auricularia heimuer*	Beijing, Miyun	2020.9.9	ON649970	ON649917
JZBQH11	*T. pholiotae*	*Pholiota adipose*	Beijing, Haidian	2020.9.25	ON649971	ON649918
JZBQH12	*T. pholiotae*	*Pholiota adipose*	Beijing, Haidian	2020.9.25	ON649972	ON649919
JZBQH13	*T. pholiotae*	*Pholiota adipose*	Beijing, Haidian	2020.9.25	ON649973	ON649920
JZBQT0Z1	*T. lentinulae*	*Lentinula edodes*	Beijing, Haidian	2021.8.3	/	/
JZBQT0Z2	*T. lentinulae*	*Lentinula edodes*	Beijing, Haidian	2021.8.3	/	/
JZBQT0Z3	*T. lentinulae*	*Lentinula edodes*	Beijing, Haidian	2021.8.3	/	/
JZBQT0Z4	*T. lentinulae*	*Lentinula edodes*	Beijing, Haidian	2021.8.3	/	/
JZBQT1Z7	*T. auriculariae*	*Auricularia heimuer*	Beijing, Tongzhou	2021.8.26	ON649949	ON649896
JZBQT1Z8	*T. auriculariae*	*Auricularia heimuer*	Beijing, Tongzhou	2021.8.26	ON649950	ON649897
JZBQT1Z9	*T. auriculariae*	*Auricularia heimuer*	Beijing, Tongzhou	2021.8.26	ON649951	ON649898
JZBQT2Z1	*T. pleuroti*	*Pleurotus ostreatus*	Beijing, Fangshan	2021.9.23	/	/
JZBQT2Z2	*T. pleuroti*	*Pleurotus ostreatus*	Beijing, Fangshan	2021.9.23	/	/
JZBQT2Z3	*T. pleuroti*	*Pleurotus ostreatus*	Beijing, Fangshan	2021.9.23	ON649974	ON649921
JZBQT2Z4	*T. pleuroti*	*Pleurotus ostreatus*	Beijing, Fangshan	2021.9.23	ON649975	ON649922
JZBQT3Z1	*T. pleuroticola*	*Pleurotus ostreatus*	Beijing, Haidian	2021.11.17	ON649976	ON649923
JZBQT3Z2	*T. pleuroticola*	*Pleurotus ostreatus*	Beijing, Haidian	2021.11.17	ON649977	ON649924
JZBQT7Z1	*T. atroviride*	*Lentinula edodes*	Hebei province, Pingquan	2022.1.1	ON649980	ON649927
JZBQT7Z2	*T. atroviride*	*Lentinula edodes*	Hebei province, Pingquan	2022.1.1	ON649981	ON649928
JZBQT7Z3	*T. atroviride*	*Lentinula edodes*	Hebei province, Pingquan	2022.1.1	ON649982	ON649929
JZBQT7Z4	*T. atroviride*	*Lentinula edodes*	Hebei province, Pingquan	2022.1.1	/	/
JZBQT7Z5	*T. atroviride*	*Lentinula edodes*	Hebei province, Pingquan	2022.1.1	/	/
JZBQT8Z1	*T. longibrachiatum*	*Lentinula edodes*	Hebei province, Pingquan	2022.1.8	ON649994	ON649941

JZBQT8Z2	*T. longibrachiatum*	*Lentinula edodes*	Hebei province, Pingquan	2022.1.8	ON649995	ON649942
JZBQT8Z3	*T. longibrachiatum*	*Lentinula edodes*	Hebei province, Pingquan	2022.1.8	/	/
JZBQT8Z4	*T. atroviride*	*Lentinula edodes*	Hebei province, Pingquan	2022.1.8	ON649983	ON649930
JZBQT8Z5	*T. atroviride*	*Lentinula edodes*	Hebei province, Pingquan	2022.1.8	ON649984	ON649931
JZBQT8Z6	*T. atroviride*	*Lentinula edodes*	Hebei province, Pingquan	2022.1.8	ON649985	ON649932
JZBQT10Z1	*T. citrinoviride*	*Lentinula edodes*	Hebei province, Pingquan	2022.2.11	/	/
JZBQT10Z2	*T. citrinoviride*	*Lentinula edodes*	Hebei province, Pingquan	2022.2.11	/	/
JZBQT10Z3	*T. citrinoviride*	*Lentinula edodes*	Hebei province, Pingquan	2022.2.11	/	/
JZBQT10Z4	*T. citrinoviride*	*Lentinula edodes*	Hebei province, Pingquan	2022.2.11	/	/
JZBQT10Z9	*T. atroviride*	*Lentinula edodes*	Hebei province, Pingquan	2022.2.11	ON649989	ON649936
JZBQT10Z13	*T. atroviride*	*Lentinula edodes*	Hebei province, Pingquan	2022.2.11	ON649990	ON649937
JZBQL45	*T. longibrachiatum*	*Lentinula edodes*	Henan province, Nanyang	2021.4.20	/	/
JZBQL46	*T. longibrachiatum*	*Lentinula edodes*	Henan province, Nanyang	2021.4.20	/	/
JZBQL50	*T. paraviridescens*	*Lentinula edodes*	Henan province, Nanyang	2021.4.20	/	/

Note: "/" indicates that the sequence is not uploaded.

Supplementary Table S2. Comparison of the morphological characteristics of *Trichoderma auriculariae* and its relatives.

**Species**	**Branching pattern**	**Phialides (μm)**	**Phialides L/W**	**Base width of Phialides (μm)**	**Conidia (μm)**	**L/W of Conidia**	**Chlamydospore (μm)**	**Reference**
T. auriculariae	Pyramidal, with opposing branches borne on a conspicuously broad spindle, less solitary	Ampulliform, sometime lageniform, 4.6–9.9 × (2.2–)2.7–3.8	1.4–3.5(–4.4)	1.4–2.7	Globose or subglobose, sometimes ellipsoidal, 2.7–3.8 × 2.3–3.1	1.0–1.3	Ellipsoide, globose or oblong, 4.6–7.5 × 3.8–6.3	This study

T. xixiacum	Pyramidal with opposing branches, less frequently solitary	Ampulliform to lageniform, (3.2–)3.5–7.0(–9.3) × (2.3–)2.6–3.3(–3.6)	(1.2–)1.5–2.5(–4)	1.6–2.2	Subglobose to globose, (2.0–)2.3–2.7(–3.0) × (1.6–)2.0–2.6(–3.0)	1.0–1.3(–1.7)	Unobserved	[1]
T. simmonsii	Pyramidal, each branch terminating in a cruciate whorl of up 1–5 phialides	Ampulliform to lageniform (4.2–)5.2–6.5 (–9.0) × (2.5–)3.0–3.7(–4.0)	(1.2–)1.5–2.4(–3.3)	1.7–2.2	Subglobose to ovoid, (2.5–)2.7–3.2(–3.7) × (2.2–)2.5–3.0(–3.5)	1.0–1.1(–1.4)	Sometimes present	[1]
T. vermifimicola	Pyramidal, the distance between branches relatively large, each branch terminating in a whorl of 2–3 phialides	Ampulliform to lageniform, (4.4–)5.0–10.5(–11.2) × (2.0–)2.5–3.0(–3.5)	(1.5–)1.8–2.8(–5.3)	1.6–2.5	Ovoid to subglobose, (2.0–)2.3–2.6(–3.0) × (1.5–)2.0–2.4(–2.8)	(1.0–)1.1–1.4(–1.7)	Unobserved	[2]

Supplementary Table S3. Comparison of the morphological characteristics of *Trichoderma miyunense* and its relatives.

Species	Branching pattern	Phialides (μm)	Phialides L/W	Base width of Phialides (μm)	Conidia (μm)	L/W of Conidia	Chlamydospore (μm)	Reference
T. miyunense	Pyramidal, multiple branches unpaired, often formed in whorls of 2–4 at the terminal of branches	Ampulliform to lageniform, (5.2–)5.6–9.7(−10.3) × 1.9–3.2(−3.7)	1.9–4.4	1.0–2.1(−2.6)	Ellipsoid, globose to subglobose, smooth, 2.2–3.4 × (1.8–)2–2.9	1−1.3(−1.4)	Unobserved	This study

T. ganodermatigerum	Typically tree-like, straight, or slightly curved, often in whorls of 3–4 divergent phialides	Lageniform, spindly, (1.1–) 2.8–12.3 (–16) × (0.2–) 1.9–3.4 (–3.6)	(1.6–) 1.7–5.9 (–7.0)	(0.2–)1.4–2.6 (–2.8)	Globose to subglobose, sometimes ellipsoid, (3.4–) 3.6–4.8 (–5.3) × (2.9–) 3.2–4.3 (–4.6)	1.1–1.5	Unobserved	[3]
T. caeruloviride	Pyramidal, paired lateral branches, with 3–4 whorls of phialides	(5.2–)5.3–12.2(–13.2) × (1.7–)2.0–2.8(–3.48)	Not described	Not described	Ellipsoidal to ovoid, smooth, 2.2–3.0(–3.2) × (1.9–)2.3–3.1(–3.4)	Not described	3.3–5.0(–6.6) × 3.0–4.6(–5.3)	[4]
T. amazonicum	Pyramidal, short base of secondary branch, 3 or more phialides per metula	Ampulliform, 6.4–7.7 × 3.3–3.5	(1.3–)1.9–2.3(–2.7)	(2.1–)2.4–2.7(–3.0)	Globose, scar generally visible, 3.2–3.4 × 3	(1.0–)1.1–1.2(–1.3)	Chlamydospore-like structures in clusters	[5]
T. pleuroti	Gliocladium-like	Ampulliform, 4.2–7 × 2–4	Not described	Not described	Ellipsoidal, 2.8–4.2 × 1.6–2.2	Not described	Yes	[5]
T. pleuroticola	Pyramidal, short base of secondary branch, 3 or more phialides per metula	Ampulliform, 5.5–11 × 3–4.2	Not described	Not described	Subglobose, broadly ellipsoidal, scar sometimes obvious, 2.5–3.5 × 2.0–2.8	1.2	Yes	[5]

T. ceratophylletum	Tree-like, often terminating in 3–5 phialides and paired branches, occasionally unilateral	Ampulliform to globose, (3.7–)4.1–8.4(–9.7) × 2.3–4.1	(1.0–)1.2–2.8(–3.2)	Not described	Ovoid, sometimes ellipsoid, smooth, 2.5–3.9 × 1.9–2.9(–3.2)	1.0–1.7	Not described	[6]
T. confertum	Tree-like, with a broad main axis, branches unpaired, rebranching up to 4 times	Ampulliform, lageniform or subulate (6.9–)8.3–12.5(–20.8) × 2.5–4.2	2.0–6.3	1.4–2.9	ellipsoid, globose to subglobose, smooth, 2.8–4.2 × 2.5–3.1	1.0–1.6	Unobserved	[7]

Supplementary Table S4. Comparison of the morphological characteristics of *Trichoderma pholiotae* and its relatives.

Species	Branching pattern	Phialides (μm)	Phialides L/W	Base width of Phialides (μ m)	Conidia (μm)	L/W of Conidia	Chlamydospo re (μm)	Reference
T. pholiotae	Pyramidal with opposing branches, with one terminal whorl of generality 3–4 phialides	Ampulliform or lageniform, (4.1−)4.9−10.9(−11 .6) × 2.4−4.2(−5.0)	1.4−3.4(−3. 9)	(1.3−)1.4−3.1 (−3.4)	Elliptic to subspheroidal, less globose, smooth, 2.6−3.8(−4.2) × 2.4−3.3(−3.5)	1−1.3	Intercalary or terminal, ellipsoide, globose, 5.0−7.4(−8.3) × (3.9−)4.9−7.0	This study
T. guizhouense	Verticillate and forming a more or less pyramidal structure	Ampulliform to lageniform, 4.5–10 × 2–3	Not described	Not described	Globose, smooth, mostly 2–3	Not described	Unobserved	[8]

T. simile	Tree-like, side branches arising from main axis asymmetrically, perpendicular to the axis	Ampulliform, less lageniform with long, (3.8–)4.3–11.9(–14.3) × (2.3–)2.7–3.9	1.3–4.4(–5.2)	1.5–2.8(–3.6)	Oval, elliptic to subspheroidal, less oblong, smooth, 2.6–3.2 × 2.2–2.8	1.0–1.2	Elliptic or round, smooth, terminal and intercalary, 4.2–7.8 × 4.0–7.2	[9]
T. asiaticum	Comprise a distinct main axis with one terminal whorl of 4–5 phialides and mostly paired side branches	Ampulliform to lageniform, (3.0–)4.0–6.0(–7.0)×(1.0–)2.0–3.0(–4.0)	(1.0–)1.3–3.0(–4.0)	Not described	(2.3–)2.4–3.0(–3.1) × (2.0–)2.1–2.7(–2.8)	(1.0–)1.1–1.3(–1.4)	Unobserved	[9]
T. pseudoasiaticum	Verticillium-like, typically with 1–3 branching levels	Ampulliform, (5.2–)6.1–9.0(–9.7)×(2.1–)2.6–3.6(–4.0)	(1.1–)1.5–3.6(–5.2)	(1.0–)1.4–2.3(–2.6)	2.4–3.2 × 2.4–3.0	1.0–1.1	Globose, smooth, terminal, 4.7–7.7×4.0–7.6	[9]

Supplementary Table S5. The growth rate of three new species in this study incubated at different temperatures and media.

	T. auriculariae			*T. miyunense*			*T. pholiotae*		
	25 °C (mm)	30 °C (mm)	35 °C (mm)	25 °C (mm)	30 °C (mm)	35 °C (mm)	25 °C (mm)	30 °C (mm)	35 °C (mm)
CMD	65–66	69–70	8–10	51–52	65–66	No growth	71–72	73–74	13–18
PDA	47–49	66–68	5–7	42–43	51–54	No growth	67–68	70–72	8–10
SNA	47–49	51–55	5–7	30–33	25–29	No growth	49–50	54–55	8–10

References

1. Chaverri, P., Branco-Rocha, F., Jaklitsch, W., Gazis, R., Degenkolb, T., Samuels, G.J. Systematics of the *Trichoderma harzianum* species complex and the re-identification of commercial biocontrol strains. *Mycologia* **2015**, *107*, 558-590.
2. Gu, X., Wang, R., Sun, Q., Wu, B., Sun, J.-Z. Four new species of *Trichoderma* in the Harzianum clade from northern China. *Mycokeys* **2020**, *73*, 109-132.
3. An, X.Y., Cheng, G.H., Gao, H.X., Li, X.F., Yang, Y., Li, D., Li, Y. Phylogenetic analysis of *Trichoderma* species associated with green mold disease on mushrooms and two new pathogens on *Ganoderma sichuanense*. *J. Fungi* **2022**, *8*, 704.
4. Rodriguez, M.D.H., Evans, H.C., De Abreu, L.M., De Macedo, D.M., Ndacnou, M.K., Bekele, K.B., Barreto, R.W. New species and records of *Trichoderma* isolated as mycoparasites and endophytes from cultivated and wild coffee in Africa. *Sci. Rep-uk.* **2021**, *11*, 5671.
5. Chaverri, P., Gazis, R.O., Samuels, G.J. *Trichoderma amazonicum*, a new endophytic species on *Hevea brasiliensis* and *H. guianensis* from the Amazon basin. *Mycologia* **2011**, *103*, 139-151.
6. Yuan, H.S., Lu, X., Dai, Y.C., Hyde, K.V.D., Kan, Y.H., Kusan, I., He, S.H., Liu, N.G., Sarma, V.V., Zhao, C.L., Cui, B.K., Yousaf, N., Sun, G.Y., Liu, S.Y., Wu, F., Lin, C.G., Dayarathne, M.C., Gibertoni, T.B., Conceicao, L.B., Garibay-Orijel, R., Villegas-Rios, M., Salas-Lizana, R., Wei, T.Z., Qiu, J.Z., Yu, Z.F., Phookamsak, R.T., Zeng, M., Paloi, S., Bao, D.F., Abeywickrama, P.D., Wei, D.P., Yang, J., Manawasinghe, I.S., Harishchandra, D., Brahmanage, R.S., De Silva, N.I., Tennakoon, D.S., Karunarathna, A., Gafforov, Y., Pem, D., Zhang, S.N., Santiago, A., Bezerra, J.D.P., Dima, B., Acharya, K., Alvarez-Manjarrez, J., Bahkali, A.H., Bhatt, V.K., Brandrud, T.E., Bulgakov, T.S., Camporesi, E., Cao, T., Chen, Y.X., Chen, Y.Y., Devadatha, B., Elgorban, A.M., Fan, L.F., Du, X., Gao, L., Goncalves, C.M., Gusmao, L.F.P., Huanraluek, N., Jadan, M., Jayawardena, R.S., Khalid, A.N., Langer, E., Lima, D.X., De Lima-Junior, N.C., De Lira, C.R.S., Liu, J.K., Liu, S., Lumyong, S., Luo, Z.L., Matocec, N., Niranjan, M., Oliveira, J.R.C., Papp, V., Perez-Pazos, E., Phillips, A.J.L., Qiu, P.L., Ren, Y.H., Ruiz, R.F.C., Semwal, K.C., Soop, K., De Souza, C.a.F., Souza-Motta, C.M., Sun, L.H., Xie, M.L., Yao, Y.J., Zhao, Q., Zhou, L.W. Fungal diversity notes 1277-1386: taxonomic and phylogenetic contributions to fungal taxa. *Fungal Divers.* **2020**, *104*, 1-266.
7. Chen, K., Zhuang, W.Y. Seven new species of *Trichoderma* from soil in China. *Mycosystema* **2017**, *36*, 1441-1462.
8. Li, Q.-R., Tan, P., Jiang, Y.-L., Hyde, K.D., Mckenzie, E.H.C., Bahkali, A.H., Kang, J.-C., Wang, Y. A novel *Trichoderma* species isolated from soil in Guizhou, *T. guizhouense*. *Mycol. Prog.* **2013**, *12*, 167-172.
9. Zheng, H., Qiao, M., Lv, Y.F., Du, X., Zhang, K.Q., Yu, Z.F. New species of *Trichoderma* isolated as endophytes and saprobes from southwest China. *J. Fungi* **2021**, *7*, 467.
